# Acute Effects of Foam Rolling and Stretching on Physical Performance and Self-Perceived Fatigue in Young Football Players

**DOI:** 10.3390/jfmk10010036

**Published:** 2025-01-17

**Authors:** Elzan Bibić, Valentin Barišić, Borko Katanić, Andrii Chernozub, Nebojša Trajković

**Affiliations:** 1 Faculty of Physical Education and Sports, University of East Sarajevo, 71000 Sarajevo, Bosnia and Herzegovina; elzanbibic9@gmail.com; 2 Faculty of Kinesiology, University of Zagreb, 10000 Zagreb, Croatia; valentin.barisic@kif.unizg.hr; 3 Montenegrin Sport Academy, 81000 Podgorica, Montenegro; borkokatanic@gmail.com; 4 The Sport and Physical Culture Theory Department, Lesya Ukrainka Volyn National University, 43025 Lutsk, Ukraine; chernozub@gmail.com; 5 Faculty of Sport and Physical Education, University of Niš, 18000 Niš, Serbia

**Keywords:** soccer, fitness, recovery, fatigue

## Abstract

**Background/Objectives**: The aim of this study was to examine the acute effects of foam rolling and traditional stretch treatments on physical performance and self-perceived fatigue in youth football players. **Methods**: The sample of participants consisted of 20 youth football players from the Serbia Under-17 league. Participants were randomly assigned to one of two groups, the Foam group (age: 16.6 ± 1.5 years) or the Stretch group (age: 16.9 ± 1.0 years), with each group consisting of 10 participants. The first measurement (I) was conducted before the match; then, the football players played the match, which was followed by the second measurement (II), and afterward, the groups performed their foam and stretch activities. The third measurement (III) was conducted 2 h after the recovery interventions, and the fourth measurement (IV) was conducted 24 h after the match. Players were tested for the following: linear sprints at 5 m, 10 m, and 20 m, squat jumps (SJs), countermovement jumps (CMJs), and arm-driven countermovement jumps (ACMJs). Participants also completed self-assessment questionnaires on fatigue (Hooper scales) and perceived exertion (Borg scales). **Results**: A 2 × 4 split-plot ANOVA revealed that there were no differences between the foam rolling and stretching treatments in any parameter of physical performance or self-perceived fatigue. However, a separate within-group analysis showed that the Foam group achieved significantly better sprint times in the third and fourth measurements (2 and 24 h post-match, respectively) compared to measurements taken immediately after the football match. Regarding self-perceived parameters, foam rolling reduced fatigue and stress at 2 and 24 h post-match. On the other hand, the stretching treatment only affected the stress parameter, which was reduced 24 h after the football match. **Conclusions**: These findings indicate that although there were no significant differences between treatments, foam rolling demonstrated certain advantages compared to stretching. Specifically, foam rolling may offer benefits in enhancing subjective recovery and reducing perceived exertion in young football players. However, these conclusions should be interpreted with caution, as the study was cross-sectional and involved a small sample of young football players.

## 1. Introduction

Football is characterized by high-intensity movements, which are crucial in executing key technical and tactical actions and reflect the overall running performance during the game [1]. According to findings from a systematic review, football involves intermittent high-intensity, short-duration movements such as sprinting, dribbling, jumping, tackling, and kicking [2]. Elite football players report using various recovery strategies following exercises and games, such as contrast water therapy, active recovery, massage, stretching, compression garments, electrical stimulation, nutrition, and hydration, as well as sleep [3]. However, it is uncertain how their subsequent outcomes could be improved with these proposed strategies.

Stretching exercises utilizing various techniques can expand a joint’s range of motion, as highlighted in a systematic review [4]. Another review also emphasizes that foam rolling, involving the use of a foam cylinder, has gained significant popularity in recent years [5]. Foam rolling has become an accepted recovery technique, helping to improve the range of motion and reduce pain following sports practice and performance [6]. Foam rolling can be considered a type of self-induced massage as the roller’s pressure on the muscles imitates physical manipulation by the individual who uses it [7].

Foam rolling workouts are essential for therapy and sports [8]. Self-massage using foam rolling is a popular intervention technique among professional athletes and recreationally active people due to its affordability, ease of use, and direct relationship with therapeutic massage, which is thought to improve performance and recovery [9]. Athletes use foam rollers to apply pressure on soft tissues, whereas roller massagers employ the upper limbs to target particular muscles [10]. Foam rolling exerts its effects through various physiological mechanisms. The mechanical pressure applied to the fascia activates intrafascial mechanoreceptors, which, via the central and autonomic nervous systems, help to reduce muscle and fascia tone [11]. Additionally, the pressure on the fascia may activate Ruffini nerve endings and interstitial tissue receptors, leading to a decrease in sympathetic tone and enhancing blood circulation and arterial function [12,13]. Together, these processes facilitate a reduction in tissue stiffness and indirectly help to more quickly remove metabolic waste products, reduce inflammation, and accelerate recovery, while also improving mobility [12].

On the other hand, it has been established in a meta-analytical review that static stretching increases the range of motion (ROM) and reduces muscle and tendon tone [14]. While some authors believe that this reduction in tone occurs because static stretching can affect sensory input by modulating supraspinal and spinal excitability, its effect on muscle activation (measured by EMG) is often contradictory [15]. In line with this, Shah et al. [14] have found that this reduction involves a decrease in the mechanical properties of muscle–tendon tissue. Still, it may not change corticospinal excitability, spinal reflex excitability, or muscle architecture parameters. These mechanisms should certainly be examined in more detail moving forward.

Previous research [16,17] found that the restoration of metabolic homeostasis, muscular damage, and anaerobic and physical performance declines after a competitive match and requires a minimum of 24–72 h of recovery, which can be lengthy when teams are in brief phases of match congestion [18]. As a result, various recovery techniques have evolved, including stretching [19] and foam rolling [20]. Studies in football have primarily focused on the effects of stretching on physical performance, particularly in sprinting [21], agility [22], and flexibility [23], as well as in injury prevention and recovery [24]. Similarly, foam rolling has been investigated for its effects on recovery and performance parameters such as agility, Total Quality Recovery (TQR), perceived muscle soreness [25], blood flow [6], bioelectric activity [26], fatigue [27], range of motion (ROM), and muscle damage [28].

Some important findings include those of Rey et al. [25], who showed that 20 min of foam rolling exercises on the quadriceps, hamstrings, adductors, glutes, and gastrocnemius had a large effect on the recovery of agility (ES = 1.06) and perceived muscle soreness (ES = 1.02). In addition, Kasahara et al. [20] reported that a 30 s intervention involving 15 foam rolls and vibration foam rolling interventions effectively increased the range of movement and pain pressure threshold and decreased tissue hardness up to 5 and 10 min after the intervention. Further, Michalski et al. [26] found that a foam rolling program affected the bioelectric activity of specified muscles in regional football players. Also, Moradi and Monazzami [29] reported that foam rolling had a positive effect on muscle recovery compared to the control group 24 h after a match, but no significant difference was found in performance when compared to the active recovery group. Regarding stretching, Doroshenko [24] highlighted its positive effects on injury prevention and quicker recovery for football players, while Erol [30] noted its benefit in increasing hamstring flexibility. However, in contrast, it has been concluded that stretching does not significantly impact agility [22] and may even have a negative effect on sprint performance [21].

Although these studies examined recovery parameters, results for both techniques remain inconsistent, suggesting the need for further investigation. Additionally, most studies focus on senior players [20,26], and there is a gap in research concerning the effects of these techniques on young football players. Since these methods are widely used in practice, it is important to determine which technique produces better acute and long-term recovery effects. It is particularly important to emphasize that there is no study directly comparing these two treatment methods, which leaves a gap in the literature regarding these treatments. Given that past research has largely analyzed these techniques separately, this study aims to compare the acute effects of foam rolling and traditional stretching on physical performance and perceived fatigue in young football players. To the authors’ knowledge, this is the first study to compare the acute effects of foam rolling and stretching on physical performance and fatigue, contributing to a better understanding of these recovery techniques. This, in turn, will help technical and medical professionals create successful recovery strategies to improve performance and reduce muscle damage.

## 2. Materials and Methods

### 2.1. Participants

The sample of participants consisted of 20 youth football players from the Serbia Under-17 league, which is a top-tier youth national football competition in Serbia. The criteria for inclusion in this study were as follows: football players from the Serbia Under-17 league, aged ≥15 to ≤18 years, with a training age of ≥5 years, and without a recent injury (>12 months) or any illness at that moment. Participants were randomly assigned to one of two groups in a 1:1 manner, while taking into consideration their playing positions. This approach was employed to ensure that positional demands specific to football were balanced between the groups. Finally, the total sample was assigned to either the Foam group or the Stretch group, with each group consisting of 10 participants. Table 1 provides detailed characteristics of the study population. All participants took part voluntarily and were informed of the purpose of this research. Since the participants are minors, their parents provided written consent for their participation in the study. Additionally, all data have been anonymized to ensure the confidentiality of the players. The research was conducted in accordance with the Helsinki Declaration, adhering to ethical standards for biomedical research involving humans. It was also approved by the Ethics Committee of the Faculty of Sport and Physical Education, University of Niš (04-651/2).

### 2.2. Procedures

**Figure 1:**
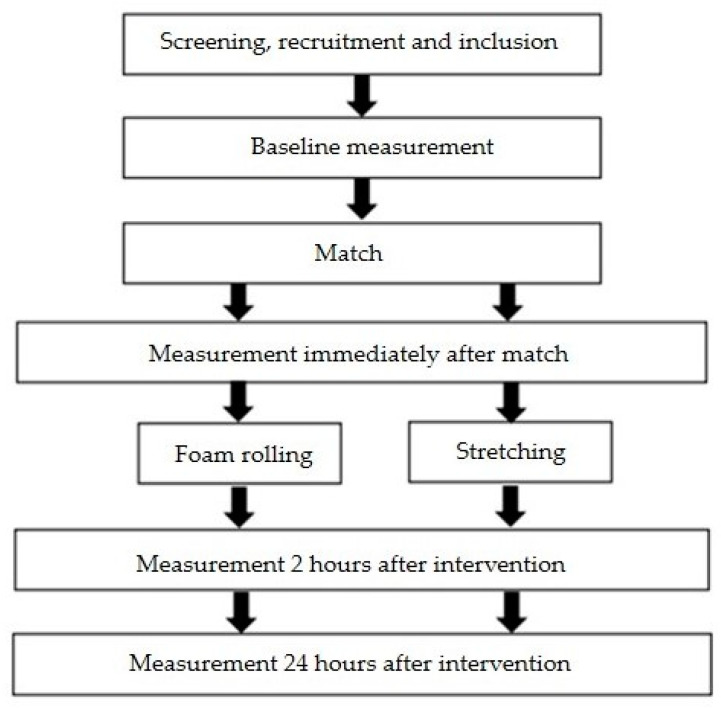
Schematic representation of experimental design.

All participants were initially assessed for anthropometric characteristics and body composition using five parameters: body height, body mass, calculated BMI, and the percentages of body fat and muscle. To examine the effects of foam and stretching training, the football players were tested at four measurement points (Figure 1) as follows: The first measurement (I) was conducted before the match, followed by the football players playing the match, after which the second measurement was taken (II), and afterward, the groups performed their foam and stretch activities. The third measurement (III) was conducted 2 h after the recovery interventions, and the fourth measurement (IV) was conducted 24 h after the match. The measurement included vertical jump tests following sprint performance over 5, 10, and 20 m.

**Figure 2:**
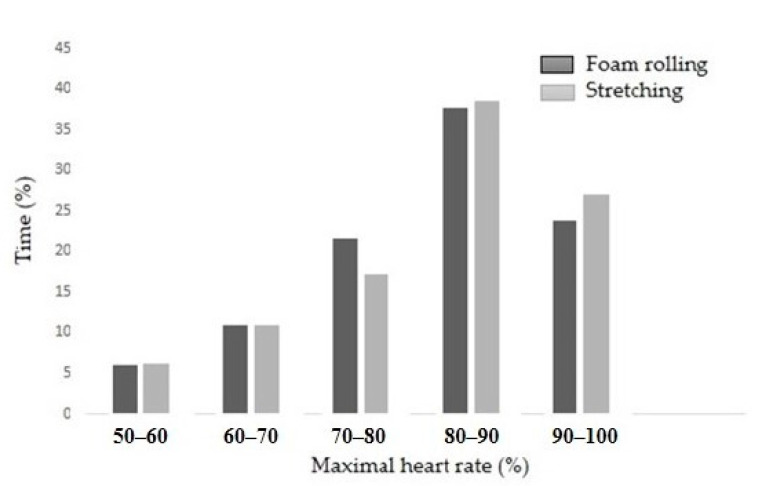
Time spent (%) in various HR zones as a percentage of maximum heart rate (HRmax) during the match.

Specifically, before the first measurement, participants completed a standardized warm-up that included moderate jogging (8 min), static stretching (5 min), and brief periods of high-intensity running (2 min). After warming up, all participants underwent the initial measurement (I) and completed a series of fitness tests consisting of 6 variables. These tests included linear sprints at 5 m, 10 m, and 20 m. The participants also completed squat jumps (SJs), countermovement jumps (CMJs), and arm-driven countermovement jumps (ACMJs). Before the initial measurements, they completed a self-assessment questionnaire on fatigue using the Hooper and Borg scales. After the initial measurements, a football match took place. In Figure 2, the results for the intensity of the match by zone for both groups are presented. The heart rate of the soccer players during the match was recorded using the Polar Team Pro (Polar Team Pro v.2.0., Kempele, Finland). The data recorded during the match were categorized into pre-determined heart rate (HR) zones using the software, with HRmax defined as the highest heart rate achieved during match play. There were no significant differences between the groups in match intensity. Additionally, the maximal heart rate reached during the game was 186 ± 3 and 184 ± 2 bts·min^−1^ in the foam rolling and stretching groups, respectively. Following the match, the participants underwent a second measurement, after which they completed 15 min of foam or stretch training, depending on their group. The third measurement point was 2 h later, and the final measurement was taken 24 h after the match. Time intervals were chosen based on existing studies, where acute effects were generally measured within 0–2 h post-activity [6,28] but also up to 24 h post-activity [28,29]. All tests were conducted in the same order. Participants were asked not to consume food or supplements for the first 2 h after the football match, with only water permitted as a drink.

### 2.3. Experimental Treatment

#### 2.3.1. Foam Training

Foam rollers measuring 45 cm in length and 15 cm in diameter and of a medium density (about 1.2 kg/m^3^) were used for the experimental treatments. The roller model used is the TriggerPoint™ GRID 1.0 Foam Roller (Durham, NC, USA) which was also used in another study [30]. The treatment protocol contained the following guidelines: (1) the foam roller is used immediately after the game; (2) session duration: 20 min; (3) the roller is used on the main muscle groups (quadriceps, back, calves, glutes); (4) each muscle group is treated for 2–3 min.

#### 2.3.2. Stretch Training

A standardized static stretching program, including guides for the correct execution of the exercises, was used to implement the experimental program in the stretching group. The modified stretching protocol was used according to protocol mentioned in an earlier study [31]. The protocol contained the following guidelines: (1) participants from the experimental group perform static stretching immediately after the match; (2) session duration: 20 min; (3) each muscle group (quadriceps, hamstrings, calves, glutes, lower back muscles) is stretched for 2–3 min per exercise; (4) each stretch exercise is held in the stretch position for 30 s, with a 10 s break between exercises. Specific stretching exercises included the following:

Quadriceps:

Standing quad stretch—participants stood upright, bent one knee, and held their ankle behind their back with the corresponding hand, keeping their torso upright.

Hamstrings:

Seated forward fold—participants sat with legs extended straight and reached forward to touch their toes, keeping the spine aligned.

Calves:

Standing calf stretch—participants stood facing a wall, stepped back with one leg, and pressed the heel into the floor while keeping the rear leg straight.

Glutes:

Supine figure-four stretch—lying on their back, participants crossed one ankle over the opposite knee and pulled the supporting leg toward their chest.

Lower Back:

Seated forward fold—sitting with legs straight, participants inhaled to lengthen the spine and then exhaled and hinged at the hips to reach forward toward their feet.

### 2.4. Measurements

#### 2.4.1. Anthropometrics and Body Composition

Body composition parameters were measured using a professional body composition analyzer, the InBody770 (InBody Co., Ltd., Seoul, Republic of Korea), which has been verified for its reliability with 95% accuracy [32]. The following variables were assessed using this instrument: body weight, body fat percentages (BF%), and muscle mass percentages (MM%). The body height of youth footballers was measured using a portable stadiometer (Seca Ltd., Bonn, Germany) with an accuracy of 0.1 cm. Body mass index (BMI) was calculated using the standard formula BMI = BW(kg)/BH(m^2^).

#### 2.4.2. Physical Performance

##### Linear Sprint Performance

The linear sprint test involves a task where players run a distance of 20 m at maximum speed. Time was recorded using electronic timing gates (Witty, Microgate, Bolzano, Italy). All results were automatically stored in the memory of the remote device. For the purposes of measurement, four pairs of photo stations were used, placed 1 m above the ground at the start line, at 5 m, at 10 m, and at the finish line (20 m). The test begins with the subject breaking the beam of a photoelectric cell at the start line and ends with the subject breaking the beam of a target photoelectric cell. In addition to the recorded time for 20 m, the splits at 5 and 10 m were also noted. Each participant repeated the test twice, with a 2 min rest between trials. The best time (the lowest final result over 20 m) was used for further analysis. Results were recorded in seconds with an accuracy of 1/100.

##### Jump Performance

To assess explosive strength based on jumping performance, three types of jumps were performed: squat jump (SJ), countermovement jump (CMJ), and arm-driven countermovement jump (ACMJ). All jumps were performed on a photocell mat (Optojump, Microgate, Bolzano, Italy). The validity and reliability of the Optojump system have been confirmed in previous research [33].

The squat jump test is executed from a squatting position (knee flexion at a 90-degree angle) without a swing of the arms. The CMJ is performed from a standing position without a swing of the arms. The ACMJ is performed from a standing position with a swing of the arms. The tests measure the maximum height of the vertical jump in centimeters.

#### 2.4.3. Self-Perceived Fatigue

##### The Hooper Questionnaire

For the self-assessment of sleep quality, fatigue, stress, and muscle soreness, the Hooper questionnaire [34] was used. This questionnaire has been used in numerous studies in football [35,36,37]. The questionnaire required participants to subjectively rate their perceived sleep quality, fatigue, stress, and muscle soreness. Participants were asked to assess how they have felt in terms of these domains. Each domain was scored on a scale from 1 to 7, with lower scores indicating more favorable conditions (e.g., better sleep quality, lower stress) and higher scores indicating less favorable conditions, with the total score indicating the athlete’s form state or readiness for training [34].

##### Borg Category-Ratio-10 Scale (BORG-CR10)

For the assessment of the rate of perceived exertion (RPE) or the subjective monitoring of exercise intensity, the Borg Category-Ratio-10 scale was used. Players were given instructions on how to use this scale [38]. Participants were instructed to focus on how difficult, strenuous, and demanding the physical task was, rather than on any sensations of pain or discomfort [39]. The ratings are arranged on a horizontal scale for the assessment of perceived exertion from 0 (“nothing at all”) to 10 (“very, very hard”).

### 2.5. Statistics

Descriptive statistics are reported as the arithmetic mean and standard deviation (mean ± SD). A repeated-measures ANOVA was used to compare differences in parameters at four different measurement points within each group separately, while a 2 × 4 split-plot ANOVA was used to determine the effects of the two experimental treatments. Statistical significance was set at *p* < 0.05. Data analysis was performed using IBM SPSS Statistics 26 software (Statistical Package for Social Sciences, v26.0, SPSS Inc., Chicago, IL, USA).

## 3. Results

### 3.1. Physical Performance

As shown in Table 2, based on the repeated-measures ANOVA test, differences were found between measurements in the 10 m sprint (*p* = 0.001) and 20 m sprint (*p* = 0.001) in the Foam group. Post hoc tests revealed that participants in the Foam group had significantly slower times in the 10 m sprint in the second measurement compared to the third and fourth measurements, as well as in the 20 m sprint in the second measurement compared to the third measurement. There were no differences in other physical performance parameters in the Foam group.

Regarding physical performance, there were no differences between different measurement points in the Stretch group.

### 3.2. Self-Perceived Fatigue

Based on the repeated-measures ANOVA test (Table 2), a significant difference was found between different measurement points for three out of five parameters, namely, stress (*p* = 0.039), fatigue (*p* = 0.01), and the exertion rating (*p* = 0.01) in the Foam group. Post hoc analysis indicated that participants in the Foam group had significantly higher values of stress, fatigue, and Borg exertion ratings in the second measurement compared to all other measurements. Additionally, for the Borg exertion rating, lower values were achieved in the third measurement compared to the fourth measurement.

As for the Stretch group, significantly higher values for stress were observed in the second measurement compared to the first and fourth measurements. Additionally, significantly lower values were reported for the parameters of fatigue and muscle soreness in the first measurement compared to all other measurements.

### 3.3. Foam vs. Stretch Group

The 2 × 4 split-plot ANOVA was used to determine the effects of the Foam and Stretch treatments. Based on the results (Table 2), it was found that there was no significant difference between the given programs in any parameter of physical performance and self-perceived fatigue.

**Table 2:** The difference between the Foam and Stretch groups of youth football players in linear sprint, jump ability, and self-perceived fatigue: repeated-measures and split-plot ANOVA.

Variables	Foam Group				F	*p*	np2	Stretch Group				F	*p*	np2	F	*p*	np2
	I	II	III	IV				I	II	III	IV						
**5 m sprint (s)**	1.08 ± 0.04	1.13 ± 0.06	1.06 ± 0.08	1.05 ± 0.08	3.043	0.10	0 .566	1.07 ± 0.04	1.12 ± 0.06	1.05 ± 0.05	1.06 ± 0.04	3.620	0 .073	0 .608	0 .512	0.67	0 .088
**10 m sprint (s)**	1.81 ± 0.08	1.86 ± 0.08 ^a,b^	1.81 ± 0.09	1.80 ± 0.09	17.741	0.01 **	0 .884	1.79 ± 0.05	1.82 ± 0.06	1.78 ± 0.06	1.81 ± 0.07	1.210	0 .374	0 .341	0 .781	0.52	0 .128
**20 m sprint (s)**	3.07 ± 0.07	3.12 ± 0.13 ^a^	3.06 ± 0.10	3.09 ± 0.08	29.418	0.01 **	0 .927	3.05 ± 0.06	3.13 ± 0.10	3.05 ± 0.09	3.10 ± 0.11	3.621	0 .073	0 .608	0 .602	0.62	0 .101
**SJ (cm)**	33.61 ± 4.20	34.70 ± 5.31	35.60 ± 4.78	34.09 ± 4.97	2.836	0.11	0 .549	33.24 ± 4.43	34.87 ± 3.37	35.26 ± 4.41	33.30 ± 3.90	2.091	0 .190	0 .473	0 .127	0.94	0 .023
**CMJ (cm)**	34.44 ± 5.17	36.77 ± 4.99	36.40 ± 5.98	37.42 ± 7.14	2.523	0.14	0 .520	35.20 ± 4.16	36.04 ± 2.91	36.80 ± 4.55	34.69 ± 5.05	0 .866	0 .502	0 .271	1.990	0.15	0 .272
**ACMJ (cm)**	40.31 ± 4.66	41.46 ± 2.80	42.81 ± 6.52	42.42 ± 6.75	0 .750	0.55	0 .243	40.70 ± 4.08	40.65 ± 4.90	41.75 ± 5.79	40.67 ± 7.31	0 .484	0 .704	0 .172	0 .438	0.72	0 .076
**Sleep**	3.20 ± 1.48	3.20 ± 1.48	3.00 ± 1.33	2.00 ± 0.94	3.059	0.10	0 .433	2.10 ± 0.74	2.10 ± 0.74	2.10 ± 0.74	2.40 ± 1.07	0 .403	0 .541	0 .043	2.643	0.10	0 .237
**Stress**	1.10 ± 0.32	2.10 ± 0.99 ^a,b,c^	1.00 ± 0.00	1.20 ± 0.42	4.879	0.03 *	0 .676	1.60 ± 0.70	2.50 ± 1.08 ^b,c^	2.20 ± 1.23	1.70 ± 0.67	5.024	0 .036 *	0 .683	1.475	0.25	0 .217
**Fatigue**	2.10 ± 1.10	4.20 ± 1.14 ^a,b,c^	2.30 ± 1.16	2.90 ± 1.20	16.326	0.01 **	0 .875	2.10 ± 1.45 ^c,d,e^	4.30 ± 1.34	3.50 ± 1.35	3.80 ± 1.37	6.074	0 .023 *	0 .722	1.371	0.28	0 .205
**Muscle soreness**	2.10 ± 0.88	3.10 ± 1.45	2.30 ± 1.16	2.70 ± 1.34	3.382	0.08	0 .592	2.30 ± 1.16 ^c,d,e^	3.50 ± 2.17	3.20 ± 1.48	3.60 ± 1.90	6.126	0 .023 *	0 .724	0 .823	0.50	0 .134
**Exertion rating**	2.00 ± 1.31	4.90 ± 1.97 ^a,b,c^	2.20 ± 1.60 ^f^	3.55 ± 2.24	16.066	0.01 **	0 .873	1.70 ± 2.62	5.10 ± 1.20	4.00 ± 1.41	4.70 ± 2.50	3.526	0 .077	0 .602	2.293	0.11	0 .301

Note: the values presented are means ± SD; SJ—squat jump; CMJ—countermovement jump; ACMJ—arm-driven countermovement jump; F—F value; *p*—statistical significance; np2—Partial Eta squared; I—initial measurement before the match; II—measurement after the match; III—measurement 2 h after the match; IV—measurement 24 h after the match; *—*p* < 0.05; **—*p* < 0.01; ^a^—2 > 3; ^b^—2 > 4; ^c^—2 > 1; ^d^—3 > 1; ^e^—4 > 1; ^f^—4 > 3.

## 4. Discussion

The main findings of this study reveal that there was no significant difference between the given treatments in any parameter of physical performance or self-perceived fatigue. However, separate analyses within the groups showed that the Foam group achieved significantly better sprint times in the third and fourth measurements (2 and 24 h after the match, respectively) compared to in the measurement taken right after the match. In the Stretch group, no differences were observed between the measurements. Regarding the self-perceived fatigue parameters (stress, fatigue, and exertion rating), the highest values were recorded immediately after the match, while the Foam treatment significantly reduced feelings of fatigue and stress 2 and 24 h after the match. This was not the case with the Stretch group, where only stress showed significantly lower values after 24 h. This indicates that although there was no significant difference between the treatments when each group was observed individually, foam rolling did show certain benefits. The lack of positive effects could be attributed to the small sample size, suggesting that future research should include a larger sample of participants.

Foam rolling has garnered significant attention as a recovery- and performance-enhancement tool in sports. The primary rationale behind foam rolling is its potential to alleviate muscle soreness and enhance muscle recovery, which, in turn, could positively influence athletic performance. Rey et al. [25] investigated the effects of foam rolling as a recovery tool among professional soccer players, reporting that foam rolling could facilitate recovery processes post-exercise by reducing muscle soreness and perceived fatigue. This suggests that foam rolling might indirectly contribute to better performance by allowing athletes to maintain higher training loads and recover more effectively between sessions. In the context of vertical jump performance, Jones et al. [40] observed that foam rolling did not produce significant improvements in vertical jump height when used in isolation. However, the study highlighted that foam rolling could potentially serve as a preparatory activity by increasing blood flow and reducing muscle stiffness, thus priming the muscles for subsequent explosive actions. Kopec et al. [41] further examined the influence of foam rolling in combination with dynamic stretching on vertical jump performance. They found that while dynamic stretching alone improved vertical jump performance, the addition of foam rolling did not confer any additional benefits. This finding aligns with the notion that foam rolling might not directly enhance explosive power but could serve other preparatory or recovery purposes. The current results indicate that while foam rolling, used as a recovery method after a soccer match, may have some positive effects on performance, it does not demonstrate significant advantages compared to other treatments. The discrepancy among studies could be due to several reasons. Recovery is influenced by multiple factors, including muscle damage, inflammation, and metabolic byproducts, which foam rolling may not adequately address. Additionally, the timing, duration, and intensity of foam rolling protocols may vary widely in studies, leading to inconsistent results. The psychological expectation of relief from soreness, often attributed to placebo effects, might also play a role, with actual physiological benefits being minimal. Furthermore, foam rolling may be less effective for highly trained athletes, whose recovery processes are already optimized through other interventions such as nutrition, hydration, and advanced recovery techniques.

On the other hand, stretching, particularly dynamic stretching, is widely accepted as a key component of warm-up routines, aiming to increase muscle temperature, flexibility, and readiness for physical activity. Dynamic stretching is often preferred over static stretching in pre-activity routines due to its ability to improve acute performance. Seçer and Kaya [42] compared the immediate effects of foam rolling followed by dynamic stretching versus dynamic stretching alone on flexibility, balance, and agility in male soccer players. Their results indicated that dynamic stretching significantly enhanced flexibility and balance, crucial components for optimizing speed and agility in soccer. The combination of foam rolling and dynamic stretching did not show superior benefits over dynamic stretching alone, suggesting that while foam rolling may aid in muscle preparation, it does not necessarily enhance the acute performance benefits derived from dynamic stretching. Moreover, Kyranoudis et al. [31] conducted a study on the acute effects of a combined foam rolling and static stretching program on hip flexion and jumping ability in soccer players. They found that the combination of foam rolling and static stretching improved hip flexion range of motion, which is beneficial for soccer-specific movements like sprinting and kicking. However, the effects on vertical jump performance were not significantly different from those observed with stretching alone, which is in line with our results. This finding suggests that while foam rolling may contribute to enhanced flexibility, it does not necessarily translate to improvements in explosive performance measures like the vertical jump. Additionally, Wiewelhove et al. [7] and Skinner et al. [43] conducted meta-analyses that further supported the notion that foam rolling can positively affect recovery and range of motion but has limited impact on acute performance outcomes such as vertical jump and speed. These reviews emphasize that while foam rolling is beneficial for recovery and flexibility, its immediate effects on performance are minimal when compared to dynamic stretching or other warm-up activities. The current body of research suggests that while foam rolling is effective for enhancing recovery and flexibility, its direct effects on vertical jump performance and speed are limited when used in isolation or even in combination with stretching.

Blades et al. [44] examined the duration of foam rolling and its effects on flexibility and vertical jump performance, finding that longer durations of foam rolling improved flexibility but did not significantly enhance vertical jump height. This finding aligns with the broader consensus that foam rolling primarily affects flexibility and recovery, with a limited direct impact on explosive power or speed.

The current results showed no significant changes in recovery following stretching modalities. Stretching may not significantly impact recovery after a match due to its limited influence on the underlying physiological processes involved in muscle recovery. While stretching can improve flexibility and reduce muscle stiffness, it does not directly address muscle damage, inflammation, or the accumulation of metabolic waste products that contribute to post-exercise fatigue [45]. Moreover, stretching primarily targets the muscle–tendon unit. Additionally, the benefits of stretching are often more associated with improving the range of motion and preventing injury rather than accelerating recovery. Variability in stretching techniques, duration, and timing across studies could also lead to inconsistent findings, with some protocols potentially being too mild to elicit a meaningful recovery response.

When the mechanisms of these two treatments are compared, certain differences are noticeable. Specifically, foam rolling focuses on the application of mechanical pressure to the fascia, which activates intrafascial mechanoreceptors, helping to reduce muscle and fascia tone, improve circulation, and decrease inflammation [11,12]. On the other hand, static stretching works by reducing muscle and tendon tone through the elongation of muscle fibers. Static stretching increases the range of motion but does not directly affect the fascia [14]. Therefore, the difference in mechanisms between these treatments is that foam rolling primarily targets the fascia and activates mechanoreceptors that influence circulation and inflammation reduction, while static stretching mainly affects muscles and tendons, enhancing flexibility and reducing tone, without a direct effect on the fascia or deeper muscle structures. However, when the acute effects of these two treatments were directly examined, no differences were found between the foam rolling and stretching treatments. Although the mechanisms behind these treatments differ, both suggest certain benefits and are used in practice. This could be one of the reasons why one group did not achieve better results than the other. Furthermore, these results pertain to acute effects, which does not exclude the possibility that differences between the treatments might emerge over a longer period.

Overall, this study represents a significant contribution to the understanding of the acute effects of these two different recovery methods in football players. The acute effects on a wide range of physical performance and self-perceived fatigue parameters were thoroughly analyzed at multiple time points. Additionally, one of the key advantages of this research is that it is the first study to compare these two recovery techniques in football players, thereby filling a gap in the existing literature. In addition to its scientific value, this study also has practical significance. It demonstrates to coaches and physiotherapists that foam rolling can be used as a tool to reduce fatigue and improve physical performance in the short term, especially during periods of intense schedules when players need to recover quickly.

The current study has some limitations. First, the sample size in our study is small, limiting the generalizability of the findings to the broader athletic population. The subjective nature of measuring recovery, often reliant on self-reported measures of soreness or fatigue, introduces potential bias and variability. Furthermore, the short-term nature of many studies does not account for the long-term effects of these interventions, which may be crucial in understanding their true impact on recovery. The lack of a standardized control group in our study may also confound results.

## 5. Conclusions

The current study, conducted on young football players, showed no significant differences between foam rolling and stretching regarding their effects on recovery when measured by physical performance outcomes such as speed and vertical jump. However, foam rolling appears to have better effects on reducing fatigue and lowering exertion ratings, indicating that young football players may feel less fatigued and perceive exercise as less strenuous after foam rolling. While the findings highlight the potential for foam rolling to be a beneficial addition to recovery routines, particularly in improving subjective recovery and reducing perceived exertion in young football players, the results should be interpreted with caution as this was a cross-sectional study with a small sample of young football players. 

## Figures and Tables

**Table 1:** Description of the sample for the Foam and Stretch groups of youth elite football players.

	Foam Group	Stretch Group
Age (number)	16.6 ± 1.5	16.9 ± 1.0
Body Height (cm)	181.4 ± 4.1	178.2 ± 6.6
Body Weight (kg)	71.0 ± 9.5	66.9 ± 6.5
BMI (score)	21.6 ± 2.6	21.1 ± 1.7
BF% (%)	12.4 ± 3.1	13.2 ± 3.9
MM% (%)	43.6 ± 1.7	43.7 ± 2.2

Note: the values presented are means ± SD; BF%—body fat percentages; MM%—muscle mass percentages.

## Data Availability

The data presented in this study are available upon request from the corresponding author.

## References

[1] Teixeira J.E., Branquinho L., Leal M., Morgans R., Sortwell A., Barbosa T.M., Monteiro A.M., Afonso P., Machado G., Encarnação S. (2024). Match-to-Match Variation on High-Intensity Demands in a Portuguese Professional Football Team. J. Funct. Morphol. Kinesiol..

[2] Stanković M., Čaprić I., Katanić B., Špirtović O., Maljanović D., Nailović H., Trajković N. (2024). Proprioceptive Training Methods (PTM) in Female Soccer Players—A Systematic Review. BMC Sports Sci. Med. Rehabil..

[3] Nédélec M., McCall A., Carling C., Legall F., Berthoin S., Dupont G. (2013). Recovery in soccer: Part II—Recovery strategies. Sports Med..

[4] Medeiros D.M., Martini T.F. (2018). Chronic effect of different types of stretching on ankle dorsiflexion range of motion: Systematic review and meta-analysis. Foot.

[5] Konrad A., Tilp M., Nakamura M. (2021). A Comparison of the Effects of Foam Rolling and Stretching on Physical Performance: A Systematic Review and Meta-Analysis. Front. Physiol..

[6] Alonso-Calvete A., Padron-Cabo A., Lorenzo-Martínez M., Rey E. (2021). Acute Effects of Foam Rolling on Blood Flow Measured by Ultrasonography in Soccer Players. J. Strength Cond. Res..

[7] Wiewelhove T., Döweling A., Schneider C., Hottenrott L., Meyer T., Kellmann M., Ferrauti A. (2019). A Meta-Analysis of the Effects of Foam Rolling on Performance and Recovery. Front. Physiol..

[8] Freiwald J., Baumgart C., Kühnemann M., Hoppe M.W. (2016). Foam-Rolling in Sport and Therapy—Potential Benefits and Risks: Part 2—Positive and Adverse Effects on Athletic Performance. Sports Orthop. Traumatol..

[9] Weerapong P., Hume P.A., Kolt G.S. (2005). The Mechanisms of Massage and Effects on Performance, Muscle Recovery, and Injury Prevention. Sports Med..

[10] Cheatham S.W., Stull K.R. (2018). Comparison of Three Different Density-Type Foam Rollers on Knee Range of Motion and Pressure Pain Threshold: A Randomized Controlled Trial. Int. J. Sports Phys. Ther..

[11] Shah S., Bhalara A. (2012). Myofascial release. Inter. J. Health Sci. Res..

[12] Schleip R. (2003). Fascial plasticity – a new neurobiological explanation: Part 2. J. Bodyw. Mov. Ther..

[13] Hotfiel T., Swoboda B., Krinner S., Grim C., Engelhardt M., Uder M., Heiss R.U. (2017). Acute effects of lateral thigh foam rolling on arterial tissue perfusion determined by spec-tral doppler and power doppler ultrasound. J. Strength Cond. Res..

[14] Shah R., Samuel M.W., Son J. (2023). Acute and chronic effects of static stretching on neuromuscular properties: A meta-analytical review. Appl. Sci..

[15] Behm D.G., Kay A.D., Trajano G.S., Blazevich A.J. (2021). Mechanisms underlying performance impairments following prolonged static stretching without a comprehensive warm-up. Eur. J. Appl. Physiol..

[16] Ispirlidis I., Fatouros I.G., Jamurtas A.Z., Nikolaidis M.G., Michailidis I., Douroudos I., Taxildaris K. (2008). Time-Course of Changes in Inflammatory and Performance Responses Following a Soccer Game. Clin. J. Sports Med..

[17] Nedelec M., McCall A., Carling C., Legall F., Berthoin S., Dupont G. (2014). The Influence of Soccer Playing Actions on the Recovery Kinetics After a Soccer Match. J. Strength Cond. Res..

[18] Arruda A.F., Carling C., Zanetti V., Aoki M.S., Coutts A.J., Moreira A. (2015). Effects of a very congested match schedule on body-load impacts, accelerations, and running measures in youth soccer players. Int. J. Sports Physiol. Perform..

[19] Marin P.J., Zarzuela R., Zarzosa F., Herrero A.J., Garatachea N., Rhea M.R., García-López D. (2012). Whole-Body Vibration as a Method of Recovery for Soccer Players. Eur. J. Sports Sci..

[20] Kasahara K., Konrad A., Yoshida R., Murakami Y., Sato S., Koizumi R., Nakamura M. (2024). Comparison of Acute and Prolonged Effects of Short-Term Foam Rolling and Vibration Foam Rolling on the Properties of Knee Extensors. Biol. Sport.

[21] Iatridou G., Dionyssiotis Y., Papathanasiou J., Kapetanakis S., Galitsanos S. (2018). Acute effects of stretching duration on sprint performance of adolescent football players. Muscles Ligaments Tendons J..

[22] Sermaxhaj S., Arifi F., Iber A., Bahtiri A., Havolli J., Sermaxhaj S. (2018). The effect of static stretching in agility and isokinetic force at football players. Sport Mont..

[23] Erol E., Yildiz R., Yildiz A., Dogan F.E., Elbasan B. (2023). Acute effects of three different stretching techniques on hamstring flexibility in professional football players. Balt. J. Health Phys. Act..

[24] Doroshenko E.Y. (2015). Application of stretching techniques in physical rehabilitation of football players with traumas of upper and lower limbs. Pedagog. Psychol. Med.-Biol. Probl. Phys. Train. Sports.

[25] Rey E., Padrón-Cabo A., Costa P.B., Barcala-Furelos R. (2019). Effects of Foam Rolling as a Recovery Tool in Professional Soccer Players. J. Strength Cond. Res..

[26] Michalski T., Król T., Michalik P., Rutkowska M., Dąbrowska-Galas M., Ziaja D., Kuszewski M. (2022). Does Self-Myofascial Release Affect the Activity of Selected Lower Limb Muscles of Soccer Players?. J. Hum. Kinet..

[27] Pahlevi R., Rusdiana A., Haryono T., Hidayat I.I., Kurniawan T. (2024). Effectiveness of massage gun and foam roller methods on fatigue recovery in football athletes. Hal. Olahraga Nusant. J. Ilmu Keolahragaan.

[28] Markovic G. (2015). Acute effects of instrument assisted soft tissue mobilization vs. foam rolling on knee and hip range of motion in soccer players. J. Bodyw. Mov. Ther..

[29] Moradi H., Monazzami A. (2020). Effects of cryotherapy and foam rolling recovery methods on performance and muscle damage indices in young male soccer players after simulated soccer match. J. Arch. Mil. Med..

[30] Ağaoğlu M.M., Usgu S., Ağaoğlu B.C., Seyhan S. (2024). Investigation of Acute Effects of Different Density Foam Roller Models on Hamstring Muscle Stiffness and Flexibility in Professional Soccer Players. Online J. Recreat. Sports.

[31] Kyranoudis A., Arsenis S., Ispyrlidis I., Chatzinikolaou A., Gourgoulis V., Kyranoudis E., Metaxas T. (2019). Acute Effects of Combined Foam Rolling and Static Stretching on Hip Flexion and Jumping Ability in Soccer Players. J. Phys. Educ. Sports.

[32] McLester C.N., Nickerson B.S., Kliszczewicz B.M., McLester J.R. (2020). Reliability of Various InBody Analyzers Compared to DXA in Healthy Men and Women. J. Clin. Densitom..

[33] Glatthorn J.F., Gouge S., Nussbaumer S., Stauffacher S., Impellizzeri F.M., Maffiuletti N.A. (2011). Validity and Reliability of Optojump Cells for Estimating Vertical Jump Height. J. Strength Cond. Res..

[34] Hooper S.L., Mackinnon L.T. (1995). Monitoring Overtraining in Athletes. Sports Med..

[35] Douchet T., Humbertclaude A., Cometti C., Paizis C., Babault N. (2021). Quantifying accelerations and decelerations in elite women soccer players during regular in-season training as an index of training load. Sports.

[36] Moalla W., Fessi M.S., Farhat F., Nouira S., Wong D.P., Dupont G. (2016). Relationship between daily training load and psychometric status of professional soccer players. Res. Sports Med..

[37] Rabbani A., Baseri M.K., Reisi J., Clemente F.M., Kargarfard M. (2018). Monitoring collegiate soccer players during a congested match schedule: Heart rate variability versus subjective wellness measures. Physiol. Behav..

[38] Borg G.A. (1998). Borg’s Perceived Exertion and Pain Scales.

[39] Marcora S.M., Goldstein E.B. (2010). Effort: Perception of. Encyclopedia of Perception.

[40] Jones A., Brown L.E., Coburn J.W., Noffal G.J. (2015). Effects of Foam Rolling on Vertical Jump Performance. Int. J. Kinesiol. Sports Sci..

[41] Kopec T.J., Bishop P.A., Esco M.R. (2017). Influence of Dynamic Stretching and Foam Rolling on Vertical Jump. Athl. Train. Sports Health Care.

[42] Seçer E., Kaya D.Ö. (2021). Comparison of Immediate Effects of Foam Rolling and Dynamic Stretching versus Only Dynamic Stretching on Flexibility, Balance, and Agility in Male Soccer Players. J. Sports Rehabil..

[43] Skinner B., Moss R., Hammond L. (2020). A systematic review and meta-analysis of the effects of foam rolling on range of motion, recovery, and markers of athletic performance. J. Bodyw. Mov. Ther..

[44] Blades C., Jones T., Brownstein C., Hicks K. (2022). The acute and delayed effects of foam rolling duration on male athletes’ flexibility and vertical jump performance. Int. J. Strength Cond..

[45] Westerblad H., Bruton J.D., Katz A. (2010). Skeletal muscle: Energy metabolism, fiber types, fatigue and adaptability. Exp. Cell Res..

